# Cardiac magnetic resonance myocardial feature tracking: feasibility for use in left ventricular non-compaction

**DOI:** 10.1186/1532-429X-15-S1-E119

**Published:** 2013-01-30

**Authors:** Ian S Stone, Redha Boubertakh, Edward J Stephenson, Filip Zemrak, Roshan Weerackody, Neha Sekhri, Mark A Westwood, Ceri Davies, Saidi A Mohiddin, Steffen E Petersen

**Affiliations:** 1 Centre for Advanced Cardiovascular Imaging, London Chest Hospital, London, UK; 2 William Harvey Research Institute, Queen Mary University of London, London, UK

## Background

Cardiac magnetic resonance (CMR) myocardial feature tracking (FT) is emerging as a sensitive and reproducible method for measuring myocardial strain parameters without the need to acquire additional images. Up until now adult CMRFT studies have primarily focussed on the reproducibility of the software,with very few studies addressing disease states beyond ischaemic cardiomyopathy. The aim of this pilot study was to assess the feasibility of cine-images derived quantitative CMR FT strain parameters to differentiate between normal individuals and patients with Left ventricular non-compaction (LVNC).

## Methods

Patients were identified retrospectively from an established clinical CMR database. 8 LVNC patients with negative invasive angiography or stress CMR myocardial perfusion imaging were compared to 21 normal controls. LVNC was defined according to the Petersen criteria,with an end-diastolic ratio of non-compacted to compacted layer (NC/C) >2.3. LV morphological and functional parameters were performed off-line on a dedicated workstation. CMR 4-chamber(4CH) and mid-ventricular short axis(SAX) cine-images were analysed in systole(S) and diastole(D) using dedicated FT software(Diogenes MRI,TomTec Imaging Systems,Munich Germany).

## Results

There was no difference in age (p=0.87) or weight (p=0.84) between LVNC and normals. LV end diastolic volumes(ml): 232±81 vs.151±45 p=0.004; LV end systolic volumes(ml): 149±87 vs.70±30 p=0.038; Mean LV ejection fractions(%): 39±10 vs.55±8 p=0.002; Significant differences were identified between LVNC vs. normals in all measures of circumferential strain(cs): ScsSAX(%) -8.1 vs.-12.3 p=0.01, DcsSAX(%) -5.6 vs.15.2 p=0.01. All radial strain(rs) in SAX and 4ch views showed either significance or trends towards significance: SrsSAX(%)14.4 vs.20.9 p=0.05, DrsSAX(%) 9.10 vs.14.0 p=0.07; Srs4CH(%) 10.1 vs.14.7 p=0.07, Drs4CH(%) 7.6 vs.13.5 p=0.04. No significant differences in longitudinal strain were seen. Where significant strain differences were seen between LVNC vs. normals, significant correlation with EF across all subjects was seen: ScsSAX r=-0.88 p<0.001, DcsSAX r=0.55 p=0.004; SrsSAX r=0.84 p<0.001; Drs4ch r=0.68 p<0.001, but within the LVNC cases alone, significant correlations with EF were only demonstrated in the SAX: ScsSAX r=0.89 p=0.03, DcsSAX r=-0.723 p=0.04; rsSAX r=0.836; p=0.01. These same three parameters also correlated with NC/C: ScsSAX r=0.622 p=0.10, DcsSAX r==0.61 p=0.11; SrsSAX r= -0.694 p=0.056.

## Conclusions

This is the first study to our knowledge that confirms the ability of CMRFT to identify abnormal rs and cs, but not longitudinal strain parameters in the presence of LV non-compaction. As expected strain correlated with EF. NC/C ratio also correlated with strain. The use of CMRFT in future studies could help establish whether cs and rs are able to differentiate between normal individuals and LVNC patients with normal EF and therefore potentially identify a prognostic marker for the development of LV dysfunction in LVNC.

## Funding

Ian Stone has received a research grant from GlaxoSmithKline

